# Enhancement of Gas Barrier Properties of Graphene Oxide/Poly (Lactic Acid) Films Using a Solvent-free Method

**DOI:** 10.3390/ma13133024

**Published:** 2020-07-06

**Authors:** Fenfen Li, Caili Zhang, Yunxuan Weng, Xiaoqian Diao, Yingxin Zhou, Xinyu Song

**Affiliations:** 1 College of Chemistry and Materials Engineering, Beijing Technology and Business University, Beijing 100048, China; 13264056919@163.com; 2 Beijing Key Laboratory of Quality Evaluation Technology for Hygiene and Safety of Plastics, Beijing Technology and Business University, Beijing 100048, China; diaoxiaoqian@btbu.edu.cn (X.D.); zhouyingxin@btbu.edu.cn (Y.Z.); songxinyu722@163.com (X.S.)

**Keywords:** gas barrier film, polylactic acid, nanocomposite, graphene oxide

## Abstract

Graphene oxide(GO)/polylactic acid (PLA) nanocomposite, prepared using a solvent-free melt mixing processing, is investigated as a potential oxygen barrier packaging film in this work. In order to disperse GO homogeneously in PLA matrix, hydrophobic silane coupling agent, i.e., γ-(2,3-epoxypropoxy)propyltrimethoxysilane (KH560), is used to modify the graphene oxide sheets. The modified GO is able to be well bonded to the PLA due to the formation of covalent bonds between the epoxy groups of KH560 and the carboxyl and hydroxyl terminal groups of PLA. Furthermore, the thermal stability of GO is enhanced due to the long alkyl side chain of KH560, which could also increase the crystallinity of PLA. As a result, the crystallinity of PLA is significantly improved because of the linear KH560 chains, which can act as nucleating agents to improve the crystallization. The KH560-GO helps to reduce the O_2_ permeability of KH560-GO/PLA composite films via a dual-action mechanism: (1) providing physical barrier due to their native barrier properties, and (2) by resulting in higher degree of crystallinity. The as-prepared KH560-GO0.75/PLA is able to exhibit ca. 33% and ca. 13% decrease in the *P*_O2_ than the neat PLA and GO0.75/PLA film, respectively. Finally, the mechanical properties and impact fractured surfaces indicate that the increase in the tensile strength and elongation at break value of KH560-GO/PLA are due to the strong interfacial adhesion and the strong bonding between the epoxy group of KH560-GO and hydroxyl and carboxyl acid terminal groups of PLA matrix.

## 1. Introduction 

Food packaging is highly essential in extending the shelf life of food, and to maintain or even enhance the food quality and consistency. Despite its importance in the food manufacturing industry, one of the key flaws in food packaging material is in its migration and permeability. This is largely due to the inadequacy of the current material in providing an impermeable barrier to gases, water vapor, or natural substances that are contained within the food being packaged [1,2]. As such, there is a significant research effort in developing enhanced performing food packaging materials based on bio-based polymer, without compromising the packed food safety and quality [3,4].

Polylactic acid (PLA) is a kind of biodegradable material with excellent performance and can be used in many fields. At present, researchers are interested in the application of PLA and its composite materials in the field of 3D printing. Kozior et al. prepared electrospinning PAN nanofiber mats on 3D printed scaffolds of rigid PLA, which have application potential in filtration [5]. Liu et al. studied 3D printing scaffolds for bone tissue repairing consisting of poly (L-lactide) (PLLA) matrix [6]. Besides the application in 3D printing, PLA is an attractive biopolymer for packaging applications. This is due to its multitude of advantages such as good biodegradability, robust mechanical properties, non-toxic nature, and high optical transparency. Hence, as a result of these advantages, PLA is often considered a promising biodegradable alternative material to replace polyethylene [7]. However, PLA is plagued with unsatisfactory gas barrier properties and poor toughness, which makes it less adopted by the packaging industry [8,9]. Thus, in order to increase the practicality of PLA film, the gas barrier performance of PLA film has to be significantly enhanced. One of the strategies to impart high barrier property to the polymer film is through the incorporation of impermeable fillers (nanoplatelets) into the polymer matrix [10,11,12,13]. This would suggest that the selection of a suitable filler is an important aspect in realizing a film with high barrier property. As a popular filler material, graphene oxide (GO) is highly attractive for its exceptional gas barrier properties due to its closely packed planar structure, substantial aspect ratio, and significantly high specific surface area. As such, recent reports on polymer-graphene oxide nanocomposites have demonstrated staggering improvements in terms of gas barrier properties as compared to their unmodified counterparts [13,14,15,16,17].

In order to leverage on the beneficial property of GO, it is highly necessary to achieve homogeneous GO dispersion in the polymer matrix so as to attain the desired properties. Three main synthesis strategies are most commonly adopted in the fabrication of GO/polymer nanocomposite; solution mixing, melt mixing, and in situ polymerization. Among these three synthesis strategies, solution mixing is widely regarded as a powerful method towards the preparation of GO/polymers because of the ease in processing GO in water or organic solvents (e.g., acetone, chloroform, tetrahydrofuran, dimethylformamide, or toluene) [13]. As such, several reports on solution mixing of GO into PLA matrix have been published in recent years. For instance, Pinto etal. incorporated different amount of GO into the PLA matrix by the solution casting method [18], while Hung et al. prepared a range of GO/PLA nanocomposites with different GO loadings using the solution mixing method [14,15]. Besides using solution mixing to incorporate GO into PLA, there have been reports on the use of the similar solution mixing method to incorporate GO into other biodegradable polymers such as poly(butylene adipate-co-terephthalate) (PBAT) and poly(vinyl alcohol) (PVA) [16,19,20]. Even though solution mixing is typically able to result in an improved dispersibility of particle in the matrix as compared to melt mixing process, particle reaggregation can still occur due to the lengthy solvent evaporation process [21]. In addition, there are several issues with the solvent mixing method such as the remaining of residual solvent in the final product, the use of excessive and costly solvents, and the generation of secondary waste stream, which have greatly hindered the mass adoption of this method in large-scale fabrication of GO/polymer packaging films [22,23].

In this work, a GO/PLA nanocomposite system fabricated from melt mixing processing is investigated for its gas barrier properties. Unlike the solvent mixing process, solvent is not required in the melt mixing process, therefore making melt mixing process a cost-effective and environmentally benign synthesis technique for large scale production. During the melt mixing process, GO is mixed with molten PLA matrix under a high shear force. More importantly, the processing effects of melt mixing and previously reported solution mixing on the dispersion and gas barrier performance of GO/PLA are compared in this work. To further increase the homogeneous GO dispersion in the PLA matrix, hydrophobic silane coupling agent, i.e., γ-(2,3-epoxypropoxy)propyltrimethoxysilane (KH560), is used. KH560 is also used to reduce and modify the graphene oxide sheets at the same time. The epoxy groups of KH560 can form covalent bonds between the carboxyl acid and hydroxyl terminal groups of PLA, which contributes to the increase in the bond strength between GO and PLA. In addition, the long alkyl side chain of KH560 could also increase the thermal stability of GO, and simultaneously increase the crystallinity of PLA.

## 2. Experimental

### 2.1. Materials

PLA was purchased from Natureworks (4032D, Mw = 17.62 × 10^4^ g mol^−1^, PDI = 2.1). To remove the moisture in PLA, it was first dried in a vacuum oven for 24 h at 60 °C. Graphene oxide, in powder form, was purchased from Yuanye Co., Ltd., Shanghai, China. Anhydrous *N*,*N*-dimethylformamide (DMF) was supplied by J&K Scientific Ltd. (Beijing, China) and dried with a 4 Å molecular sieve. Both silane coupling agent, i.e., γ-(2,3-epoxypropoxy)propyltrimethoxysilane (KH560), and ethanol were obtained from Beijing Innochem Co., Ltd. (Beijing, China).

### 2.2. Preparation of GO-KH560

The typical preparation of KH560-modified GO (KH560-GO) is illustrated below: 2 g of GO powder was firstly dispersed in 1 L distilled water, and the GO-water mixture was stirred for 5 h at 30 °C to obtain a black suspension. At the same time, a homogeneous KH560 solution was prepared by dispersing KH560 (10 mL) in an ethanol/distilled water mixture (45/5 mL) under magnetic stirring. Next, the GO suspension prepared earlier was heated at 80 °C and KH560 solution was added to the GO solution dropwise. After that, the solution was further stirred for 10 h at 80 °C to ensure the completion of the reaction. The solution was later cooled to ambient temperature, and subsequently filtered. The residue was washed with ethanol for several times, and later dried in a vacuum oven overnight at 60 °C. The dried sample collected was 2.5 g KH560-GO.

### 2.3. Film Preparation

All the films presented in this work were prepared using the melt mixing method. In order to ensure all chemicals are dried before mixing, both PLA and GO (KH560-GO for the synthesis of KH560-GO/PLA) were vacuum dried at 60 °C for 24 h before using. Haake Rheometer was used to prepare all the samples under 70 rpm and at 180 °C for ca. 5 min. The mixture consisting of PLA and GO with different weight ratios was first premixed in the chamber. This process was continued until the torque stabilized (ca. ∼2 min). The completion of dynamic vulcanization was indicated when the melt torque leveled off, after which the melting mixed samples were taken out of the cavity of the internal mixer and then the samples were left to cool to ambient temperature. A range of GO/PLA and KH560-GO/PLA samples were prepared with different amounts (0 to 0.25, 0.50, 0.75, 1.0, and 1.5 wt%) of GO and KH560-GO, respectively. To achieve the final GO/PLA and KH560-GO/PLA nanocomposite films, the samples were molded into 60–90 μm-thick films using a hydraulic press under 10 MPa at 180 °C.

To facilitate the calculation process, GO weight content in the nanocomposite was changed to volume content, using the following equation proposed by Chen et al. [24]:

(1) $$\phi_{S}=\frac{1}{1+(\rho_{S}(1-M_{S})/\rho_{P}M_{S})}$$

where $\phi_{S}$ is the volume fraction of the GO, $\rho_{S}$ is the density of GO, $\rho_{P}$ is the density of PLA, and $M_{S}$ is the mass fraction of the GO. For the conversion of GO weight content in the nanocomposites to volume content, densities of 1.25 and 1.80 g/cm^3^ for PLA and GO are used, respectively.

### 2.4. Characterization and Measurement

Thermogravimetric analysis (TGA) of GO and KH560-GO was performed with a thermogravimetry analyzer (STA-7200, HITACHI, Japan) in an N_2_ environment under a ramping rate of 10 °C/min. Raman spectrometer (HORIBA JOBIN YVON, France) was used to obtain the Raman spectra of the samples. The atomic force microscopic (AFM) (NanoNavi Station/E-sweep, Seiko Instruments, Inc., Chiba, Japan) was used to conduct the AFM analysis by using a silicon cantilever probe in a tapping mode. To prepare the sample for AFM analysis, diluted GO and KH560-GO aqueous suspension are spin-coated onto a silicon wafer. X-ray diffraction (XRD) was performed on the samples using an X-ray diffractometer (Bruker D8 Advance, Bruker Daltonics Inc., Radeberg, Germany) with a Cu radiation source operating at 40 kV and 40 mA. Differential scanning calorimetry (DSC) measurements were conducted using a Q20 differential scanning calorimeter (TA Instrument, New Castle, DE, USA). In the preparation prior to the DSC measurements, the samples were first loaded in sealed aluminum pans. During the DSC measurements, the samples were initially heated from 30 °C to 190 °C with a ramping rate of 10 °C/min. The samples were later cooled from 190 °C to 30 °C at a rate of 20 °C/min. The samples were heated from 30 °C to 190 °C at a heating rate of 10 °C/min for a second cycle. By integrating the melting enthalpy (are under the melting peaks), the heat of fusion could then be calculated. The degree of crystallinities (*χ*_c_) of the pure PLA and the composites was calculated according to Equation (2):

(2) $$\chi_{c}=\frac{\left(\Delta H_{m}-\Delta H_{cc}\right)}{w\Delta H_{m}^{0}}\times 100\%$$

where $\Delta H_{m}$ is the melting enthalpy obtained experimentally (J/g), *w* is the weight fraction of PLA in the nanocomposite. $\Delta H_{m}^{0}$ is the melting enthalpy of 100% crystalline PLA i.e., 93.7 J/g.

Tensile properties of the samples were tested using a universal testing machine (Shenzhen Labsans Material Testing Co., Ltd., Shenzhen, China) at a speed of 5 mm/min, according to the ASTM D882 standard. The dimensions of the samples used was 100 mm × 10 mm with thickness between 60–90 um. For all tests, average values for at least five specimens are recorded. Phenom Pro machine (Phenom World, Eindhoven, Netherlands) was used for the scanning electron microscopy (SEM) analysis of the impact fractured surfaces of GO/PLA and KH560-GO/PLA nanocomposite specimens, under an acceleration voltage of 10 kV.

### 2.5. Oxygen Permeation Analysis 

Oxygen permeation analysis of the nanocomposite films was conducted according to the ASTM D3985 standard by using an oxygen transmission rate tester 31M (Labthink, Jinan, China) at 23 °C with a coulometric sensor [25]. The film with an effective area of 10 cm^2^ was placed in the test chamber whereby it separates the chamber into a penetrant chamber with relative humidity of 30% and a carrier chamber with relative humidity of 0%. The purity of O_2_ used in this test was more than 99.99%. The diffusion cell was subsequently purged with an N_2_ carrier gas. At either end of the sample film, the gases, i.e., oxygen at one end and nitrogen at the other end, were under identical absolute pressures and similar flow rates. Due to the concentration gradient, oxygen can permeate from the side with high concentration into the oxygen-deficient region by penetrating through the film. To measure the oxygen concentration, the carrier gas passes through a coulometric oxygen sensor after leaving the test chamber. When the coulometric sensor has determined that the oxygen flux has stabilized, oxygen transmission is then considered to have reached steady state. The flow rate of the carrier gas is used in the calculation of the oxygen transmission rate and oxygen permeability. The permeation area of the film and the oxygen concentration in the carrier gas are directly given by the oxygen transmission testing system. Each test was repeated three times, and average oxygen permeability was obtained.

## 3. Results and Discussion

### 3.1. Structure and Morphology Analysis of GO and KH560-GO Powder

**Figure 1:**
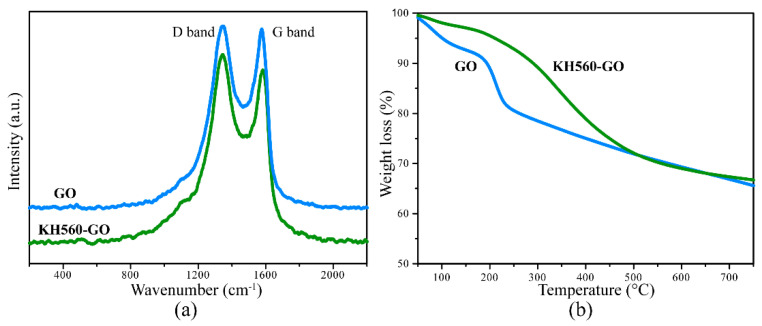
(**a**) Raman spectra and (**b**) TGA profiles of GO powder and GO-KH560 powder.

The Raman spectrum of GO, presented in Figure 1a, reveals two peaks located at 1344 and 1582 cm^−1^ which are attributed to the D and G bands, respectively. As reported by Liu et al., D band mainly represents defects such as grain boundaries, vacancies, and amorphous carbon species, while G band represents first order scattering of E_2g_ phonon of the sp^2^ C atoms [26]. The Raman spectrum of KH560-GO is also shown in Figure 1a. It can be observed that after modifying GO with the silane coupling agents, D band appears to be more prominent for KH560-GO as compared to its unmodified counterpart. As a result of the sp^3^ defects within the sp^2^ carbon framework in GO, this leads to an increase in the intensity of D band for KH560-GO. Thus, based on the Raman result, the presence of alkyl moiety within the sp^2^ carbon network of GO can be verified. Such result suggests the successful reaction between the coupling agent and the hydroxyl group of GO.

TGA is conducted for both GO and KH560-GO, and the results are shown in Figure 1b. The TGA profile of GO reveals the decomposition of the residual water molecules in between the GO sheets at temperature below 100 °C, thereby suggesting a low thermal stability for GO. As the temperature is increased to the range of 150 and 250 °C, there is rapid weight loss for GO. Such drastic weight loss could be mainly due to the removal of the oxygen functional groups present in GO [27,28]. On the other hand, the TGA profile of KH560-GO shows a much lower weight loss of 5% at a temperature of 200 °C as compared to 10% for unmodified GO at the same temperature. This phenomenon may suggest that most of the labile oxygen containing functional groups of GO are reacted with silane coupling agents. As the temperature increases to the range of 200 and 400 °C, there is a 20% weight loss in KH560-GO, which could be related to the decomposition of epoxy groups presented in KH560. The further decomposition of KH560-GO of about 31% weight loss is observed at temperatures beyond 400 °C, which may be due to the degradation of the alkyl chain of KH560.

**Figure 2:**
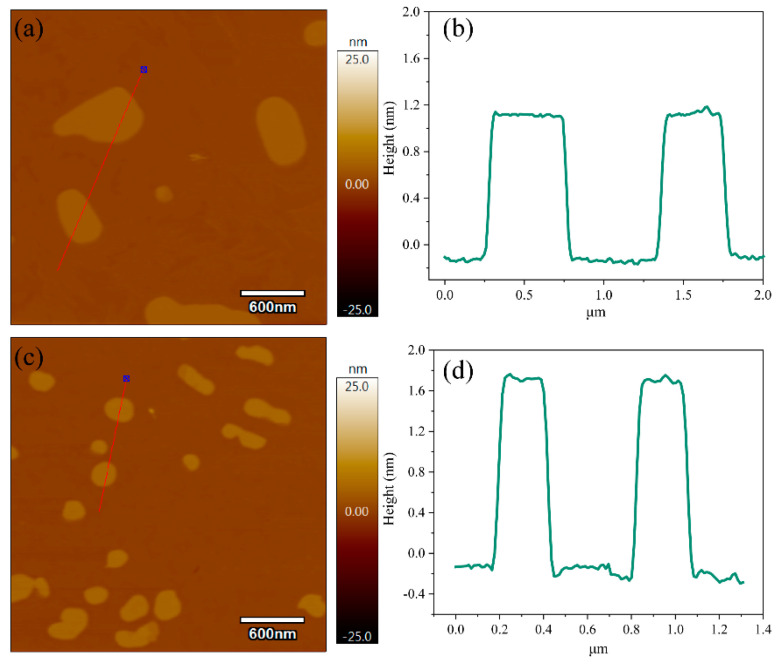
AFM image and height profiles of GO (**a**,**b**) and KH560-GO (**c**,**d**).

To investigate the thickness of the synthesized sample, AFM is conducted for both GO and KH560-GO, and their AFM topographic images and height profiles are shown in Figure 2. Based on Figure 2a,b, it can be concluded that GO is ca. 0.4–0.6 μm in size, and it possesses a thickness of ca. 1.0 nm which is approximately the thickness of a single layer GO [27,29,30]. On the other hand, KH560-GO is 0.2–0.3 μm in size with a thickness of ∼1.7 nm as shown in Figure 2c,d. The increase in thickness can be attributed to the immobilization of KH560 molecules onto the GO surface. Furthermore, there is a size reduction for KH560-GO when compared to GO. This could be due to the strong mechanical agitation during the synthesis process.

### 3.2. XRD

**Figure 3:**
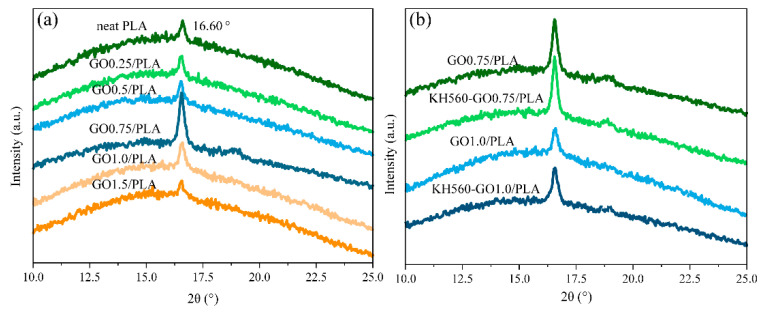
XRD curves of (**a**) GO/PLA and (**b**) KH560-GO/PLA nanocomposites.

To investigate the crystallinity and crystal structure of the polymer/GO nanocomposite, XRD was conducted on the samples so as to identify the nanocomposite structure [16,31]. Figure 3a shows the XRD spectra of neat PLA and PLA/GO nanocomposites prepared with varying amounts of GO. It can be observed that the main characteristic diffraction peak for neat PLA is located at 20 = 16.6°, which corresponds to the (110)/(200) diffraction peak [32,33]. As the GO loading is increased, the intensity of the (110)/(200) diffraction first rises and then subsequently decreases. As a result, the nanocomposite with 0.75 wt% GO loading possesses higher crystallinity as compared to the rest of the GO/PLA nanocomposites in this investigation. This result could be due to the ability of GO nanosheets to act as a nucleating agent at a lower loading level, and therefore increasing the crystallinity of the PLA [34] However, as the GO loading increases beyond 0.75 wt %, the large presence of GO in the polymer matrix could impede the regular arrangement of PLA molecular chains and thus lead to a lower crystallinity for the PLA matrix [14,15] This is due to the reduction in the surface area as a result of GO sheets restacking in GO/PLA at higher GO loadings. The XRD spectrums of the various KH560-GO/PLA, shown in Figure 3b, suggest that the crystallinity of KH560-GO/PLA is higher than that of GO/PLA. This result suggests that the presence of short KH560 chains could improve the crystallinity of PLA. In order to further investigate and verify the crystallinity of GO/PLA and KH560-GO/PLA nanocomposites, DSC was conducted.

### 3.3. DSC

**Figure 4:**
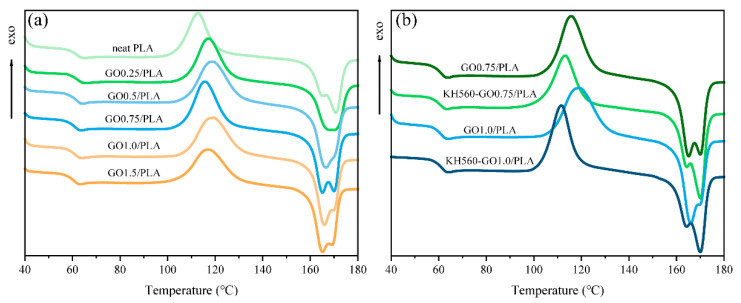
Second heating curves of (**a**) GO/PLA and (**b**) KH560-GO/PLA nanocomposites.

The nanoplates in a nanocomposite are able to lower gas permeability via two key methods: (1) by acting as a physical barrier, and (2) by enhancing the crystallinity of the polymer matrix [35,36]. The second heating DSC curves of the neat PLA, PLA/GO, and PLA/KH560-GO are shown in Figure 4. The glass transition temperature (*T*_g_), crystallinity (*χ*_c_), melting temperature (*T*_m_), and cold crystallization temperature (*T*_cc_) are summarized in Table 1. As shown in Figure 4a, with the increase in the GO content, both the *T*_g_ and crystallinity of the PLA matrix are slightly modified, while *T*_cc_ is shifted to a higher temperature. The crystallinity of PLA increases from 1.23% to 2.77% after introducing 1.5 wt % GO into the PLA. Based on these results, crystallinity of PLA is not affected much when a small amount of GO is added. After adding 0.75 wt % KH560-GO into PLA, it can be observed from Figure 4b that the crystallinity increases from 1.23% to 7.49%. As the amount of KH560-GO added to PLA increases to 1.0 wt % (KH560-GO1.0/PLA), the crystallinity of the PLA matrix decreases. However, the crystallinity of KH560-GO1.0/PLA remains higher as compared to its unmodified counterpart, i.e., GO1.0/PLA. Thus, even though long aliphatic chains in KH560 can act as heterogeneous nucleating sites, which can enhance the crystallizibility of PLA, large amounts of KH560-GO (>1.0 wt %) can have a detrimental effect on PLA crystallization. This is largely due the crosslinking formed after the reaction between the epoxy groups of KH560 with carboxyl and hydroxyl terminal groups of PLA, which leads to the restriction in the PLA chain movement and therefore reduces its crystallinity [37]. Thus, this DSC measurement result agrees well with the earlier mentioned XRD result regarding the crystallinity of PLA in the nanocomposites. The crystallization ability of KH560-GO is better than that of GO due to the long aliphatic chains of KH560. However, it is shown that excessive KH560-GO can restrict the movement of the PLA chain, which reduces its crystallinity.

### 3.4. Gas Barrier Properties of GO/PLA and KH560-GO/PLA Nanocomposite Films

**Figure 5:**
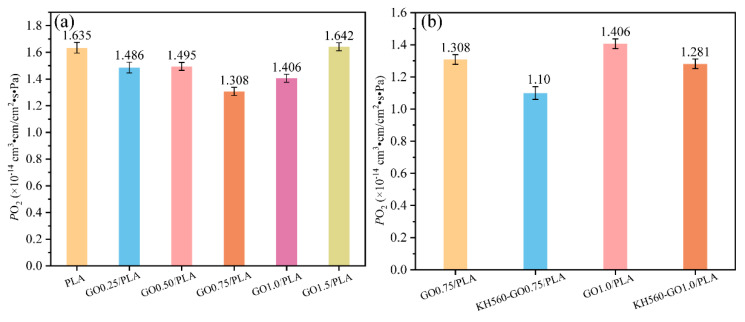
O_2_ permeability of (**a**) GO/PLA and (**b**) KH560-GO/PLA samples as a function of GO and KH560-GO content.

Table 2 summarizes the O_2_ permeability (*P*_O2_) of GO/PLA and KH560-GO/PLA nanocomposite films. To provide a more visual representation of *P*_O2_ comparison of neat PLA and its composites, a graph was plotted; it is presented in Figure 5. According to the results, GO0.75/PLA film (containing 0.75 wt % GO) shows an O_2_ permeability of 0.174 Barrer, which is a 20% reduction in permeability as compared to the neat PLA film. However, as the GO content is higher than 0.75 wt %, *P*_O2_ of the GO/PLA film increases. It can be observed that the *P*_O2_ of GO1.5/PLA is close to that of the neat PLA film. Such behavior could be a result of the poor dispersibility of GO in the PLA matrix, which in turn leads to the formation of defects in the films. The non-uniform GO platelets dispersion could be due to the high viscosity of the molten PLA. Such non-uniform dispersion phenomenon has also been reported in several papers, such as styrene–butadiene rubber (SBR) and acrylonitrile butadiene rubber (NBR) melt mixing with graphite powder [38,39,40,41,42]. Furthermore, according to the TGA results, GO shows ~10% weight loss at 200 °C, which suggests the thermal instability of the chemical structure of GO. As such, this translates to a difficulty in fabricating thermally stable GO/PLA nanocomposite films using the melt mixing method. Therefore, most GO/polymer nanocomposites (including two previously reported GO/PLA nanocomposites) are prepared with the solution mixing method instead [13,14,18]. While the solution mixing method is generally successful in preparing thermally stable GO/polymer nanocomposites, the use of a huge volume of solvent and its accompanying environmental pollution greatly hinders the practicality of this technique in large-scale production [22].

Interestingly, the KH560-GO/PLA film exhibits lower O_2_ permeability than the GO/PLA film, as shown in Table 2 and Figure 5b. For instance, the O_2_ permeability of KH560-GO0.75/PLA (0.147 Barrer) is ca. 33% and ca. 13% lower than the neat PLA and GO0.75/PLA film, respectively. However, as the amount of KH560-GO increases from 0.75 wt % to 1.0 wt %, *P*_O2_ of KH560-GO1.0/PLA (0.171 Barrer) is higher as compared to KH560-GO0.75/PLA. The higher *P*O2 in KH560-GO1.0/PLA is due to the non-uniform dispersion of excess KH560-GO in the highly viscous molten PLA during the melt mixing process.

Enhancing the oxygen barrier properties of GO/polymer composites is strongly dependent on nanosheets, crystallinity of the polymer matrix, and the interface of GO/polymer. In this work, KH560-GO helps to reduce the O_2_ permeability of KH560-GO/PLA composite films via a dual-action mechanism: (1) providing physical barrier due to their native barrier properties, and (2) by resulting in higher degree of crystallinity. Furthermore, KH560 can encourage GO to be better dispersed in the PLA matrix by compatibilizing GO and PLA.

The barrier properties of GO/PLA films prepared using the solution method by Pinto et al. [18] and Huang et al. [14] are compared and the data summarized in Table 2. The GO/PLA nanocomposite film prepared via the melt mixing method generally shows a relatively lower oxygen barrier property as compared to those prepared via the solution mixing method. Such a result is largely due to the nonuniform dispersion, as mentioned earlier.

**Figure 6:**
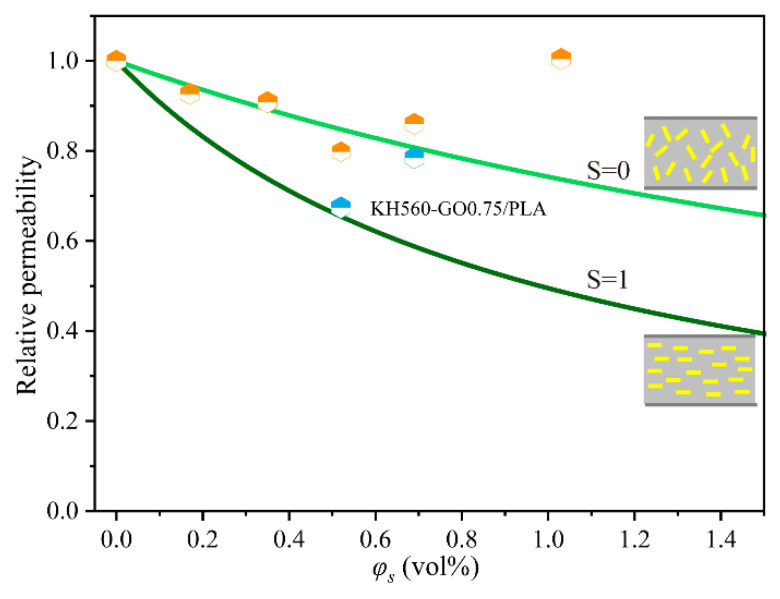
Experimental data about *P*_O2_ and the Bharadwaj model for the relative permeability in terms of GO and KH560-GO loadings are compared (the yellow symbols refer to GO/PLA and the blue symbols refer to KH560-GO/PLA).

To further investigate the impact of graphene oxide sheets on the barrier properties of GO/PLA and KH560-GO/PLA nanocomposite films, the estimated reduction in relative permeability (*R_P_*) and Bharadwaj model are compared in Figure 6 [35,43,44]. Relative permeability can be calculated using Equations (3) and (4) [45]:

(3) $$R_{P}=\frac{P_{S}}{P_{P}}=\frac{1-\phi_{S}}{1+\frac{L}{2W}\phi_{S}\left(\frac{2}{3}\right)\left(S+\frac{1}{2}\right)}$$

(4) $$S=\frac{1}{2}\left(3\cos^{2}\theta -1\right)$$

where *P*_s_ is the permeability of the nanocomposite film, *P*_p_ is the permeability of the neat PLA, $\phi_{S}$ is the volume fraction of GO, and L/W is the aspect ratio. L/W, which is the ratio of the width to the thickness of the impermeable flakes, can be determined from AFM. Thus, based on the statistical analysis of AFM images, the L/W of GO is ~200. *S* is the order parameter and it depends on the orientation of nanoplatelets, where *θ* is the angle between the plane of nanoplatelets and perpendicular to the diffusive gas molecules.

The relative permeabilities *P*_s_/*P*_p_ are calculated from oxygen permeability as a function of *ϕ*_s_ using the Bharadwaj model (*S* = 0, *L/W* = 200) and the results are plotted in Figure 6. In this investigation, due to the random dispersion of GO and KH560-GO in the PLA matrix, the experimental data related to the decrease in relative permeability are in good proximity to the predicted Bharadwaj values, when *S* = 0.

### 3.5. Mechanical Properties

**Figure 7:**
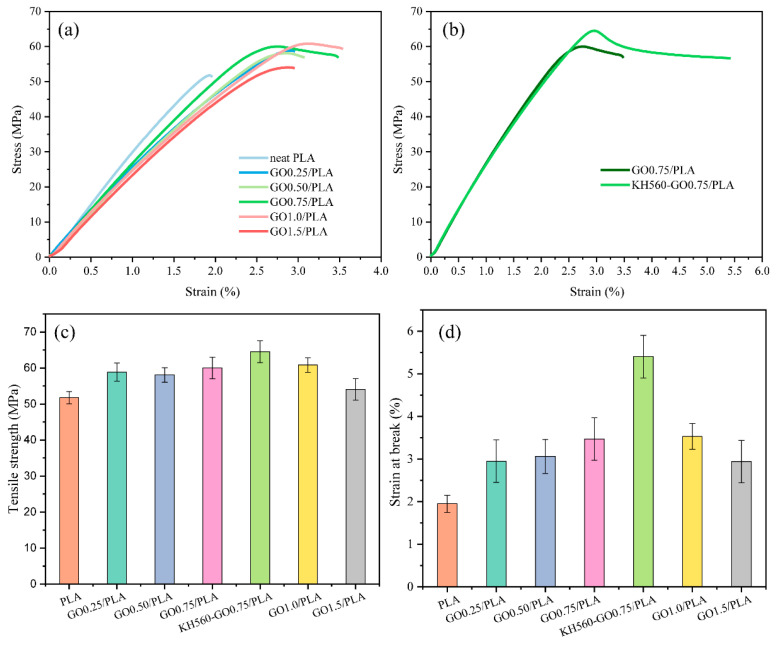
(**a**) stress-strain curves of GO/PLA nanocomposites, (**b**) comparison of stress-strain curves of GO0.75/PLA and KH560-GO0.75/PLA, (**c**) tensile strength and (**d**) elongation at break of GO/PLA and KH560-GO/PLA nanocomposites.

Mechanical properties of GO/PLA and KH560-GO/PLA nanocomposites are tested and the respective stress-strain curves, tensile strength, and elongation at break are shown in Figure 7. As the amount of GO increases from 0.25 wt % to 1.5 wt %, the tensile strength and elongation at break of GO/PLA nanocomposites increase initially and then decrease. The two main factors in determining the reinforcing efficiency of GO in composite is the dispersibility of GO in the composite and the interface interaction. The increase in GO mass fraction leads to the reducing dispersibility of GO in the PLA matrix. This poor dispersibility, in turn, results in certain degree of agglomeration, which ultimately reduces the mechanical properties of GO/PLA nanocomposites. In contrast, the tensile strength and elongation at break of KH56-GO0.75/PLA are recorded as 64.5 MPa and 5.4%, respectively. It can be concluded that KH56-GO0.75/PLA is able to demonstrate 25% enhancement in the tensile strength and 177% enhancement in the elongation at break than the neat PLA. These increase in the mechanical properties (in terms of tensile strength and elongation at break) of KH560-GO0.75/PLA is due to the strong interfacial adhesion and the strong chemical bonding between the epoxy group of KH560-GO and hydroxyl and carboxyl acid terminal groups of the PLA matrix.

### 3.6. Morphology of GO/PLA and KH560-GO/PLA Blend Films

**Figure 8:**
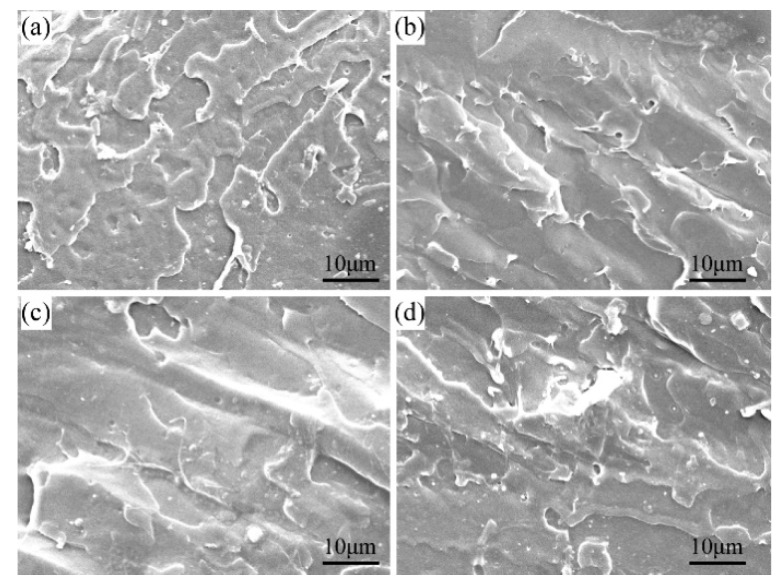
SEM images of (**a**) neat PLA, (**b**) GO0.25/PLA, (**c**) GO0.75/PLA, and (**d**) GO1.0/PLA.

To reveal the morphologies of the GO/PLA nanocomposites with varying GO content and KH560-GO0.75/PLA nanocomposite, SEM was employed. The impact fractured surfaces of the neat PLA, GO0.25/PLA, GO0.75/PLA, and GO1.0/PLA film are shown in Figure 8. The fractured surfaces of the GO/PLA nanocomposites under SEM reveal features of brittle failure. The neat PLA, GO0.25/PLA, and GO0.75/PLA show a smooth surface. On the other hand, GO1.0/PLA exhibits a rough surface with some observable agglomerates, which indicates relatively poor interfacial adhesion between PLA and the excess GO.

**Figure 9:**
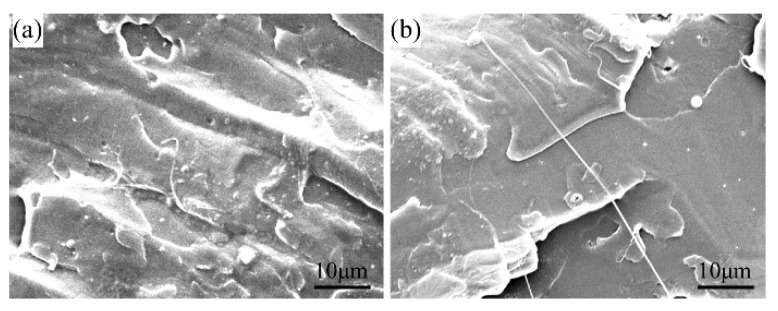
SEM images of (**a**) GO0.75/PLA and (**b**) KH560-GO0.75/PLA.

For KH560-GO0.75/PLA, some filaments can be seen on the fractured surface, as shown in Figure 9b. This is due to the toughness fracture during impact process, which shows that KH560-GO in the PLA matrix is able to exhibit more toughness effect as compared to the unmodified GO. Therefore, it can be concluded that the overall fracture mode of KH560-GO0.75/PLA is a mixture of ductile and brittle.

## 4. Conclusions

In this work, GO is modified with KH560 silane coupling agent to achieve two main objectives; (1) improve the dispersibility of GO in the PLA matrix, and (2) increase the crystallinity of PLA. The structure and morphology of the GO powder and KH560-GO powder are confirmed using Raman spectroscopy, TGA, and AFM measurements. A range of GO/PLA and KH560-GO/PLA nanocomposites are prepared via melt mixing technique and their barrier properties are investigated for potential application as oxygen barrier materials. XRD and DSC are used to evaluate the crystallinity of the nanocomposites. Due to the availability of linear KH560 chains as nucleating agent, the crystallinity of PLA is significantly improved. The KH560-GO helps to reduce the O_2_ permeability of KH560-GO/PLA composite films via a dual-action mechanism; (1) providing physical barrier due to their native barrier properties, and (2) by resulting in higher degree of crystallinity. The as-prepared KH560-GO0.75/PLA is able to exhibit ca. 33% and ca. 13% decrease in the *P*_O2_ than the neat PLA and GO0.75/PLA film, respectively. Finally, the mechanical properties and impact fractured surfaces indicate that the increase in the tensile strength and elongation at break value of KH560-GO/PLA are due to the strong interfacial adhesion and the strong bond between the epoxy group of KH560-GO and hydroxyl and carboxyl acid terminal groups of the PLA matrix.

## Figures and Tables

**Table 1:** The list of thermal properties of GO/PLA and KH560-GO/PLA nanocomposites obtained from DSC measurement.

	*T*_g_ (°C)	*T*_cc_ (°C)	Δ*H*_cc_ (J/g)	*T*_m1_ (°C)	*T*_m2_ (°C)	Δ*H*_m_ (J/g)	*χ*_c_
neat PLA	58.98	113.75	23.67	163.13	170.8	24.81	1.23
GO0.25/PLA	59.11	117.13	28.99	168.45		30.56	1.69
GO0.5/PLA	58.59	118.80	31.99	166.59	-	33.91	2.06
GO0.75/PLA	58.20	115.60	30.61	166.39	-	32.56	2.09
KH560-GO0.75/PLA	57.81	112.10	25.56	164.33	170.05	32.53	7.49
GO1.0/PLA	58.37	119.17	32.47	165.97	-	34.38	2.05
KH560-GO1.0/PLA	58.50	111.47	27.88	164.27	170.04	31.39	3.77
GO1.5/PLA	58.02	117.12	29.35	165.24	169.34	31.93	2.77

**Table 2:** Gas permeability of graphene oxide/PLA nanocomposite films.

Samples	GO Loading		Preparation Method	Permeability		Reduction
	wt %	vol %		Barrer *	10^−14^ cm^3^ cm/cm^2^ s Pa	
PLA	/	/	Melt	0.218	1.635	/
GO0.25/PLA	0.25	0.17	Melt	0.202	1.486	9
GO0.5/PLA	0.50	0.35	Melt	0.198	1.495	9
GO0.75/PLA	0.75	0.52	Melt	0.174	1.308	20
KH560-GO0.75/PLA	0.75	0.52	Melt	0.147	1.100	33
GO1.0/PLA	1.0	0.69	Melt	0.187	1.406	14
KH560-GO1.0/PLA	1.0	0.69	Melt	0.171	1.281	22
GO1.5/PLA	1.5	1.03	Melt	0.219	1.642	0
PLA [18]	-		solution	0.501	3.76	-
	0.2		solution	0.179	1.34	64
	0.4		solution	0.164	1.23	67
	0.6		solution	0.199	1.49	60
PLA [14]	-		solution	0.278	2.087	-
	0.25		solution	0.206	1.545	26
	0.5		solution	0.184	1.381	34
	1.0		solution	0.180	1.348	35
	2.0		solution	0.153	1.145	45

*1 Barrer = 1 × 10^−10^ cm^3^(STP) cm/cm^2^ s cmHg = 7.5 × 10^−18^ m^2^(STP)/s Pa = 7.5 × 10^−14^ cm^3^(STP) cm/cm^2^ s Pa; 1 Pa = 7.5005 × 10^−4^ cmHg.

## References

[1] Arvanitoyannis I.S., Bosnea L. (2014). Migration of substances from food packaging materials to foods. Crit. Rev. Food Sci..

[2] Robertson G. (2016). Structure and related properties of plastics polymers. Food Packaging: Principles and Practice.

[3] Nesic C., Castillo P., Castaño G., Cabrera-Barjas J. (2020). Bio-based packaging materials. Biobased Products and Industries.

[4] Muñoz-Bonilla A., Echeverria C., Sonseca Á., Arrieta M.P., Fernández-García M. (2019). Bio-based polymers with antimicrobial properties towards sustainable development. Materials.

[5] Kozior T., Mamun A., Trabelsi L., Wortmann M., Sabantina L., Ehrmann A. (2019). Electrospinning on 3D printed polymers for mechanically stabilized filter composites. Polymers.

[6] Liu K., Li W.Y., Chen S.T., Wen W., Lu L., Liu M.X., Zhou C.R., Luo B.H. (2020). The design, fabrication and evaluation of 3D printed gHNTs/gMgO whiskers/PLLA composite scaffold with honeycomb microstructure for bone tissue engineering. Compos. B Eng..

[7] Youssef A.M., El-Sayed S.M. (2018). Bionanocomposites materials for food packaging applications: Concepts and future outlook. Carbohydr. Polym..

[8] Lehermeier H.J., Dorgan J.R., Way J.D. (2001). Gas permeation properties of poly (lactic acid). J. Membr. Sci..

[9] Auras R.A., Singh S.P., Singh J.J. (2005). Evaluation of oriented poly (lactide) polymers vs. existing PET and oriented PS for fresh food service containers. Packag. Technol. Sci..

[10] Cui Y., Kumar S., Kona B.R., van Houcke D. (2015). Gas barrier properties of polymer/clay nanocomposites. RSC Adv..

[11] Tan B., Thomas N.L. (2016). A review of the water barrier properties of polymer/clay and polymer/graphene nanocomposites. J. Membr. Sci..

[12] Wolf C., Angellier-Coussy H., Gontard N., Doghieri F., Guillard V. (2018). How the shape of fillers affects the barrier properties of polymer/non-porous particles nanocomposites: A review. J. Membr. Sci..

[13] Cui Y., Kundalwal S., Kumar S. (2016). Gas barrier performance of graphene/polymer nanocomposites. Carbon.

[14] Huang H.-D., Ren P.-G., Xu J.-Z., Xu L., Zhong G.-J., Hsiao B.S., Li Z.-M. (2014). Improved barrier properties of poly (lactic acid) with randomly dispersed graphene oxide nanosheets. J. Membr. Sci..

[15] Huang H.-D., Zhou S.-Y., Zhou D., Ren P.-G., Xu J.-Z., Ji X., Li Z.-M. (2016). Highly Efficient “Composite Barrier Wall” Consisting of Concentrated Graphene Oxide Nanosheets and Impermeable Crystalline Structure for Poly (lactic acid) Nanocomposite Films. Ind. Eng. Chem Res..

[16] Ren P.-G., Liu X.-H., Ren F., Zhong G.-J., Ji X., Xu L. (2017). Biodegradable graphene oxide nanosheets/poly-(butylene adipate-co-terephthalate) nanocomposite film with enhanced gas and water vapor barrier properties. Polym. Test..

[17] Chen J.-T., Fu Y.-J., An Q.-F., Lo S.-C., Zhong Y.-Z., Hu C.-C., Lee K.-R., Lai J.-Y. (2014). Enhancing polymer/graphene oxide gas barrier film properties by introducing new crystals. Carbon.

[18] Pinto A.M., Cabral J., Tanaka D.A.P., Mendes A.M., Magalhaes F.D. (2013). Effect of incorporation of graphene oxide and graphene nanoplatelets on mechanical and gas permeability properties of poly (lactic acid) films. Polym. Int..

[19] Liu H., Bandyopadhyay P., Kim N.H., Moon B., Lee J.H. (2016). Surface modified graphene oxide/poly (vinyl alcohol) composite for enhanced hydrogen gas barrier film. Polym. Test..

[20] Huang H.-D., Ren P.-G., Chen J., Zhang W.-Q., Ji X., Li Z.-M. (2012). High barrier graphene oxide nanosheet/poly (vinyl alcohol) nanocomposite films. J. Membr. Sci..

[21] Zhang C., Li P., Cao B. (2015). Electrospun microfibrous membranes based on PIM-1/POSS with high oil wettability for separation of oil–water mixtures and cleanup of oil soluble contaminants. Ind. Eng. Chem. Res..

[22] Sengupta R., Bhattacharya M., Bandyopadhyay S., Bhowmick A.K. (2011). A review on the mechanical and electrical properties of graphite and modified graphite reinforced polymer composites. Prog. Polym. Sci..

[23] Kang H., Zuo K., Wang Z., Zhang L., Liu L., Guo B. (2014). Using a green method to develop graphene oxide/elastomers nanocomposites with combination of high barrier and mechanical performance. Compos. Sci. Technol..

[24] Chen B., Evans J.R. (2006). Nominal and effective volume fractions in polymer—clay nanocomposites. Macromolecules.

[25] (2010). ASTM D-3985 Standard Test Method for Oxygen Gas Transmission Rate Through Plastic Film and Sheeting Using a Coulometric Sensor. https://www.gbpitester.com/polyester-reference-material_p69.html?gclid=EAIaIQobChMIld6Ct_uw6gIVAu7tCh2AbgEbEAAYASAAEgL2WvD_BwE.

[26] Liu H., Kuila T., Kim N.H., Ku B.-C., Lee J.H. (2013). In situ synthesis of the reduced graphene oxide–polyethyleneimine composite and its gas barrier properties. J. Mater. Chem. A.

[27] Hou Y., Wang D., Zhang X.-M., Zhao H., Zha J.-W., Dang Z.-M. (2013). Positive piezoresistive behavior of electrically conductive alkyl-functionalized graphene/polydimethylsilicone nanocomposites. J. Mater. Chem. C.

[28] Park S., An J., Potts J.R., Velamakanni A., Murali S., Ruoff R.S. (2011). Hydrazine-reduction of graphite-and graphene oxide. Carbon.

[29] Wang Y., Wang F., Wang H., Song M. (2017). Graphene oxide enhances the specificity of the polymerase chain reaction by modifying primer-template matching. Sci. Rep..

[30] Chatterjee N., Eom H.-J., Choi J. (2014). A systems toxicology approach to the surface functionality control of graphene–cell interactions. Biomaterials.

[31] Mahmood H., Pegoretti A., Brusa R.S., Ceccato R., Penasa L., Tarter S., Checchetto R. (2020). Molecular transport through 3-hydroxybutyrate co-3-hydroxyhexanoate biopolymer films with dispersed graphene oxide nanoparticles: Gas barrier, structural and mechanical properties. Polym. Test..

[32] Lotz B., Li G., Chen X., Puiggali J. (2017). Crystal polymorphism of polylactides and poly (Pro-alt-CO): The metastable beta and gamma phases. Formation of homochiral PLLA phases in the PLLA/PDLA blends. Polymer.

[33] Kovalcik A., Pérez-Camargo R.A., Fürst C., Kucharczyk P., Müller A.J. (2017). Nucleating efficiency and thermal stability of industrial non-purified lignins and ultrafine talc in poly (lactic acid)(PLA). Polym. Degrad. Stabil..

[34] Xu J.-Z., Zhang Z.-J., Xu H., Chen J.-B., Ran R., Li Z.-M. (2015). Highly enhanced crystallization kinetics of poly (l-lactic acid) by poly (ethylene glycol) grafted graphene oxide simultaneously as heterogeneous nucleation agent and chain mobility promoter. Macromolecules.

[35] Tenn N., Follain N.G., Soulestin J.R.M., Crétois R.L., Bourbigot S., Marais S.P. (2013). Effect of nanoclay hydration on barrier properties of PLA/montmorillonite based nanocomposites. J. Phys. Chem. C.

[36] Cocca M., Di Lorenzo M.L., Malinconico M., Frezza V. (2011). Influence of crystal polymorphism on mechanical and barrier properties of poly (l-lactic acid). Eur. Polym. J..

[37] Lv H., Song S., Sun S., Ren L., Zhang H. (2016). Enhanced properties of poly (lactic acid) with silica nanoparticles. Polym. Adv. Technol..

[38] Yang J., Tian M., Jia Q.X., Zhang L.Q., Li X.L. (2006). Influence of graphite particle size and shape on the properties of NBR. J. Appl. Polym Sci..

[39] Ismail M., Khalaf A. (2011). Styrene–butadiene rubber/graphite powder composites: Rheometrical, physicomechanical, and morphological properties. J. Appl Polym Sci..

[40] Song S.H., Jeong H.K., Kang Y.G., Cho C.T. (2010). Physical and thermal properties of acid-graphite/styrene-butadiene-rubber nanocomposites. Korean J. Chem. Eng..

[41] Sadasivuni K.K., Ponnamma D., Thomas S., Grohens Y. (2014). Evolution from graphite to graphene elastomer composites. Prog. Polym. Sci..

[42] Zhan Y., Wu J., Xia H., Yan N., Fei G., Yuan G. (2011). Dispersion and exfoliation of graphene in rubber by an ultrasonically-assisted latex mixing and in situ reduction process. Macromol. Mater. Eng..

[43] Gao W., Alemany L.B., Ci L., Ajayan P.M. (2009). New insights into the structure and reduction of graphite oxide. Nat. Chem..

[44] Chien A.T., Lin K.F. (2007). Morphology and permeability of exfoliated PVAc-MMT nanocomposite films cast from soap-free emulsion-polymerized latices. J. Polym. Sci. A Polym. Chem..

[45] Bharadwaj R.K. (2001). Modeling the barrier properties of polymer-layered silicate nanocomposites. Macromolecules.

